# Tick infestation of birds in grasslands experiencing woody plant encroachment in the United States Great Plains

**DOI:** 10.1093/jme/tjaf072

**Published:** 2025-06-20

**Authors:** Tucker C Taylor, Jozlyn D Propst, Bruce H Noden, Scott R Loss

**Affiliations:** Department of Entomology and Plant Pathology, Oklahoma State University, Stillwater, OK, USA; Department of Entomology and Plant Pathology, Oklahoma State University, Stillwater, OK, USA; Department of Entomology and Plant Pathology, Oklahoma State University, Stillwater, OK, USA; Department of Natural Resource Ecology and Management, Oklahoma State University, Stillwater, OK, USA

**Keywords:** birds, Eastern redcedar, woody plant encroachment, landscape epidemiology, ticks

## Abstract

Woody plant encroachment is one of the largest threats to grasslands of the US Great Plains. Its spread, mainly due to fire suppression, affects entire ecosystems, including arthropod vectors, bird communities, and the ecology of vector-borne disease. Eastern redcedar (*Juniperus virginiana*), one of the primary encroaching species in this region, is known to increase abundance of pathogen-carrying tick species such as *Amblyomma americanum*; however, the role birds play in carrying ticks in association with eastern redcedar encroachment is unknown. In areas of Oklahoma representing 3 stages of eastern redcedar encroachment, we mist-netted birds, sampled larval and nymphal ticks from them, and evaluated tick infestation of birds from May to October 2023. Of 140 birds sampled, 25.7% were infested with ticks, a higher prevalence of infestation than in most previous studies of birds in the United States. Notably, some birds were infested with *Ixodes scapularis*, which has rarely been found on wildlife other than reptiles in the southern US. There were no significant differences in prevalence or intensity of tick infestation in birds across differing levels of eastern redcedar encroachment, indicating that a high proportion of birds carry ticks in all encroachment stages. This study provides the first evidence that birds contribute to the ecology of tick-borne disease systems in grasslands experiencing woody plant encroachment. Our results, which suggest birds are likely moving ticks into, out of, and among eastern redcedar-encroached grasslands of the US Great Plains, can help inform land management and public health efforts seeking to reduce disease risk.

## Introduction

Vector-borne pathogens cause some of the most frequent and potent diseases of humans and wildlife worldwide. In the United States, tick-borne pathogens like *Rickettsia*, *Ehrlichia*, and *Borrelia* contribute to >75% of vector-borne disease cases in humans (Rosenberg 2018, CDC 2022). Vector-borne disease transmission is influenced by interactions among vectors, hosts, and pathogens within their surrounding environment (ie the nidus of infection) (Reisen 2010). The effects of human-caused ecological changes on vector-borne pathogen transmission are well known, inextricably linking land cover and wildlife hosts to disease risk (Lambin et al. 2010, Guo et al. 2019, Diuk-Wasser et al. 2021). However, across large areas of the interior of North America, including the Great Plains of the United States, there has been little research evaluating how large-scale environmental changes affect the nidus of infection for tick-borne diseases, including the role of wildlife in carrying ticks and tick-borne pathogens.

In the US Great Plains, a major form of land cover change likely affecting tick-borne diseases is woody plant encroachment (WPE). WPE, the expansion of trees and shrubs into grassland and savannah ecosystems (Briske 2017), is occurring worldwide as a result of many factors including fire suppression, urbanization, landscape fragmentation, and climate change (Briggs et al. 2005, Eldridge et al. 2011, Archer et al. 2017, Wilcox et al. 2018). WPE drastically changes abiotic conditions like hydrology, solar radiation, temperature, humidity, and wind, as well as plant and wildlife diversity and wildlife habitat use (Ge and Zou 2013, Archer et al. 2017, Briske 2017). Landscape-level changes in forest cover can alter tick populations by influencing abundance and species diversity of bloodmeal hosts, which respond to landscape-scale patterns of habitat availability and fragmentation (Ogrzewalska et al. 2011, Perez et al. 2016, Cumbie et al. 2021, Diuk-Wasser et al. 2021). Therefore, WPE is likely providing suitable habitat for ticks and their hosts, increasing the presence of vector-borne diseases in encroached areas (Noden et al. 2021, Loss et al. 2022). However, no studies have evaluated the effects of WPE on the role of wildlife as hosts for ticks and tick-borne pathogens.

Although birds are not as well studied as other tick bloodmeal hosts like mammals, many studies in the United States and worldwide illustrate that birds contribute to maintenance and spread of diseases by transporting ticks infected with pathogens (Ogrzewalska et al. 2011, Hamer et al. 2012, Cumbie et al. 2021, Tardy et al. 2021) and by serving as competent reservoir hosts themselves (Comstedt et al. 2006, Ebani et al. 2021, Becker et al. 2022, Defaye et al. 2023). Further, research indicates birds are tick bloodmeal hosts and can disperse ticks from local to hemispheric scales through long distance migration (Allan et al. 2010, Cohen et al. 2015, Karim et al. 2024). An important factor influencing the contribution of birds to these disease systems is life history, as ground-foraging birds tend to have higher tick loads than other species, likely due to their tendency to occur in areas with high numbers of foraging ticks (Mitra et al. 2014, Loss et al. 2016, Heller et al. 2019, Roselli et al. 2022).

Eastern redcedar (*Juniperus virginiana*, hereafter ERC), a tree species widely encroaching into grasslands of the US Great Plains, is likely affecting tick-borne diseases, including the role of birds in carrying ticks and tick-borne pathogens (Wang et al. 2018, Fogarty et al. 2022). ERC encroachment drastically changes most aspects of grassland ecosystems (Ratajczak et al. 2012, Kishawi et al. 2023, Neumann et al. 2025), including by increasing shade and relative humidity, conditions favorable to ticks (Noden and Dubie 2017, Noden et al. 2021). Because bird community composition also changes with ERC encroachment (Chapman et al. 2004, Coppedge et al. 2004, Frost and Powell 2011), it is likely that bird interactions with ticks similarly change. Nevertheless, no studies have examined how WPE, including by ERC specifically, affects bird–tick interactions, including the prevalence and intensity of tick infestation of birds.

To address this research gap, we sampled ticks from wild birds caught in sites capturing 3 stages of ERC encroachment in Oklahoma, USA. Having one of the highest rates of ERC encroachment in the United States (Zou et al. 2018), Oklahoma is an ideal location to conduct this research. Further, research indicates ERC encroachment in Oklahoma is increasing abundance of multiple tick species, especially *Amblyomma americanum* (Noden et al. 2021, Propst 2025). Our objectives were to: (i) describe the prevalence of tick infestation (ie number of birds carrying at least one tick divided by total birds sampled) and intensity of tick infestation (ie average number of ticks on infested birds) in ERC-encroached grasslands; and (ii) analyze whether there are significant differences in prevalence and intensity of tick infestation among stages of ERC encroachment. Related to objective 2 and based on previous research suggesting increased abundance of ticks under ERC trees (Noden and Dubie 2017, Noden et al. 2021), we hypothesized that both prevalence and intensity of tick infestation of birds would increase with each successive stage of ERC encroachment, from areas that are primarily grass-covered with small and scattered ERC trees to closed-canopy ERC forest. This study will increase understanding of the effects of human-caused land cover change on vector-borne disease risk and provide land managers and public health officials with information to manage tick-borne disease risk in areas experiencing WPE.

## Methods and Materials

### Site Selection

As part of a broader study evaluating the effects of ERC encroachment on ticks and tick-borne pathogens, we identified areas in western Oklahoma that could accommodate clusters of 4 study sites which met the following criteria: (i) each site was >2 ha (large enough to contain the number of tick traps and flagging transects needed for a companion study), (ii) all 4 sites were within ~5 km of each other (to allow for sampling all 4 sites for ticks during one morning in the companion study), (iii) the 4 sites captured varying levels of ERC encroachment including open grasslands with no ERC (notably, these control grassland sites were used for companion studies of ticks but not for this study, as described below); and (iv) all sites were publicly accessible (eg state parks/wildlife management areas; properties owned by Oklahoma State University). We identified these general areas using ESRI ArcGIS (ESRI 2024) and the Oklahoma Ecological Systems Mapping (OESM; Diamond and Elliot 2015) data layer, a 10 × 10 m resolution land cover layer that covers all of Oklahoma and includes specific designation of land cover types for which eastern redcedar is a dominant species. Specifically, we considered candidate areas if they contained substantial coverage of OESM land cover categories that were likely to capture open grasslands for our control sites (207-Central Mixedgrass Prairie: Prairie/Pasture), as well as ERC-encroached areas (15005-Canyon: Juniper Shrubland; 2515-High Plains: Bottomland Eastern Redcedar Woodland and Shrubland; 2503-High Plains: Bottomland Hardwood—Eastern Redcedar Forest; 2715-High Plains: Riparian Eastern Redcedar Woodland and Shrubland; 2703-High Plains: Riparian Mixed Hardwood—Eastern Redcedar Woodland; 9115-Ruderal Eastern Redcedar Woodland and Shrubland; 14815-South Central Interior: Bottomland Eastern Redcedar Woodland and Shrubland; 15115-South Central Interior: Riparian Eastern Redcedar Woodland and Shrubland; 1815-Southeastern Great Plains: Bottomland Eastern Redcedar Woodland and Shrubland; 1915-Southeastern Great Plains; Riparian Eastern redcedar Woodland and Shrubland).

**Fig. 1.**
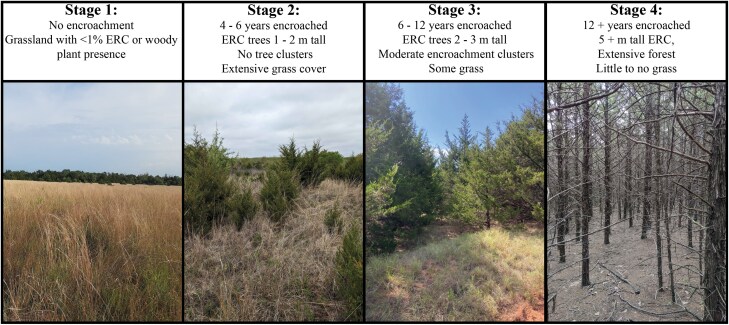
Characteristic locations from our study depicting successive stages of ERC encroachment into grasslands. Stage 1 represents un-encroached grasslands, with stages 2, 3, and 4 having progressively taller and greater coverage of ERC culminating in mature forest with virtually no grassland remaining. Photos taken in Oklahoma, USA.

**Fig. 2.**
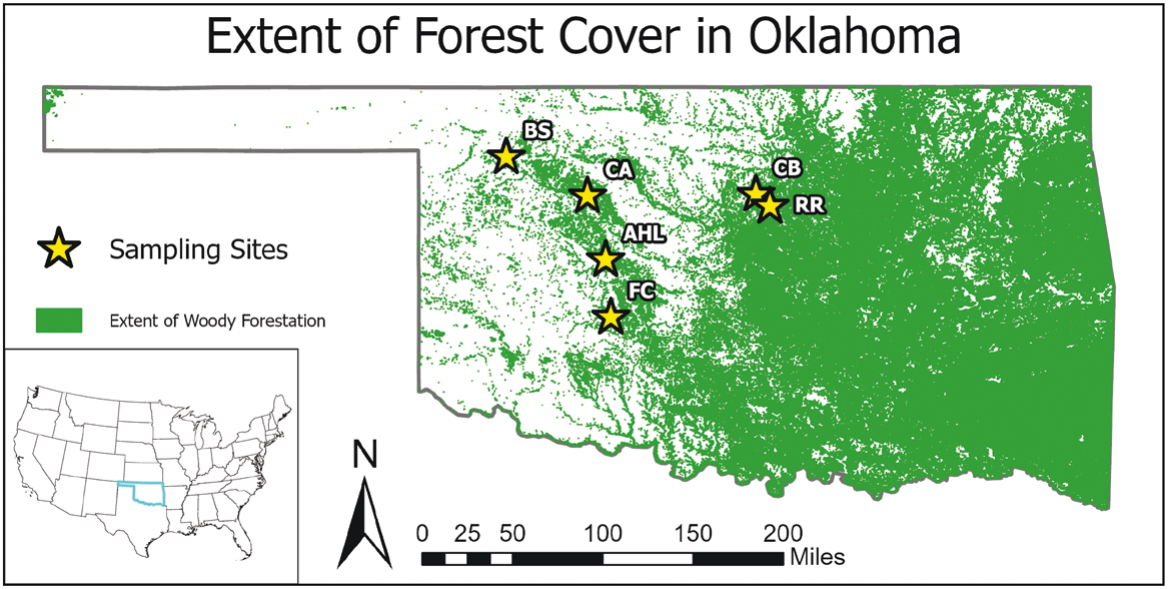
Location of 7 site clusters in Oklahoma in which we sampled birds to evaluate tick infestation prevalence and intensity (FC includes both the Fort Cobb East and Fort Cobb West site clusters); shading indicates forest cover, including eastern redcedar and all other forms of woody cover. Forest layer obtained from National Land Cover Database land cover raster (USGS and MRLC 2022). Map produced using ArcGIS Pro 3.1.0 2023.

For all candidate areas identified using the above process, we ground-truthed land cover types during field visits. We excluded areas that lacked adequate road access, that had different land covers than indicated in the OESM layer (including areas where ERC had been cut/removed), and that could not accommodate 4 sites capturing the stages of ERC encroachment illustrated and described in Fig. 1 (eg due to no or limited coverage of one or more ERC stages). For 7 remaining areas meeting criteria (Fig. 2), hereafter referred to as site clusters, we visually identified and georeferenced boundaries of 4 sites capturing distinct stages of ERC encroachment (ie 28 total sites, with 4 in each of the 7 site clusters).

Although we sought to separate all sites within clusters by at least 1.0 km, replication constraints related to logistics and areal coverage and location of encroachment stages resulted in some sites being as close as 0.11 km to each other. The average minimum distance between sites within clusters was much greater at 0.82 km (se ± 0.2 km), but we provide further discussion of the implications of site spacing in the discussion. The 4 ERC encroachment stages illustrated and described in Fig. 1 appear to be associated with varying tick abundances based on a pilot study (Noden and Dubie 2017, Noden et al. 2021). Notably, although we included all 4 stages in our companion studies focused on tick abundance and prevalence of pathogens in ticks, in this study, we only sampled birds from a subsample of 3 sites each in stages 2 to 4 (ie 9 total sites) due to the difficulty of capturing a large sample of birds in open grasslands (see additional details under “Capturing Birds and Sampling Ticks”).

The study area captured by our 7 site clusters has a continental temperate climate, and primary land covers include grassland, cropland, and forest (Wang et al. 2018). Precipitation in this study area varies along a strong west-to-east gradient, with annual averages ranging from ~420 mm/yr in the west to 780 mm/yr in the east (PRISM Climate Group 2023). Further site description is included in Table 1.

**Table 1.** General description of sites sampled during the field study in Oklahoma, USA from May to October 2023. Precipitation data were obtained from Oregon State University PRISM Climate Group (2023)

Site cluster (west to east)	Management	Yearly avg rainfall in mm (2023)	Description of surrounding landscape (non-ERC tree covers excluded from our sites)
Boiling Springs	Oklahoma Tourism and Recreation Department	499.3	Mixed ERC and oak forest with mowed and burned stage 1
Canton Wildlife Management Area	Army Corps of Engineers/WMA	568.4	Predominantly ERC, located along reservoir, grasslands used for hunting/ disc golf
American Horse Lake	Oklahoma Department of Wildlife Conservation	660.6	Predominantly ERC around small lake, hilly, exposed rock and sandy soil, patches of sumac at stages 1 and 2
Fort Cobb East and Fort Cobb West	Oklahoma Tourism and Recreation Department	666.8	Predominately ERC with patchy mixed deciduous and sumac interspersed among grassland located along reservoir
Lake Carl Blackwell	Oklahoma State University	708.9	Stages 2 to 4 entirely ERC, close proximity to seasonal stream, surrounded by private and research farmland, wild animals like boar frequently observed
Research Range	Oklahoma State University	830.3	ERC cross-timber and rangeland used by cattle, site 2 roughly 4km away from cluster

### Measurement of Tree Height and Cover

**Fig. 3.**
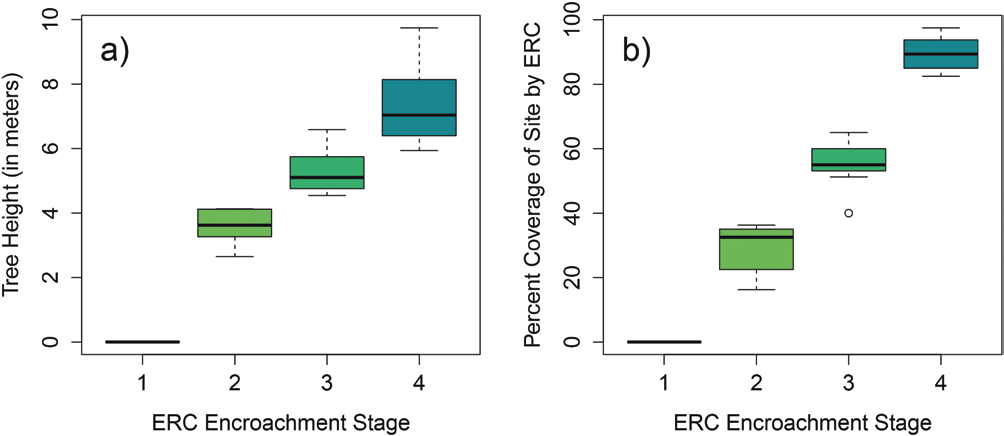
Comparison of average (a) eastern redcedar tree (ERC) height and (b) horizontal percent cover across 4 stages of ERC encroachment used for a broader study of ERC effects on ticks and tick-borne pathogens, including the 3 stages (2, 3, and 4) used in this study. All measurements were collected in Oklahoma, USA in May to June 2024.

Due to the temporal discrepancy between the timing of ground-truthing visits in early 2022 and data collection, which occurred in 2023 as described below, ERC height and spatial coverage were greater than originally planned at some sites by the end of the study period (ie some sites assigned to 1 encroachment stage during ground-truthing appeared closer to the subsequent stage by the end of sampling in 2023). In order to determine if our sites still captured distinctive stages of ERC encroachment during field sampling, we measured tree height and horizontal coverage in early 2024 at all 28 sites used in the broader study, including at the 9 sites used for this study. To measure average tree height, we took measurements at three plots at each site cluster, one at each stage (stages 2, 3, and 4), with plots centered on one tree that we used for tick CO_2_ trapping in a companion study (Propst 2025). For ERC height measurements, one tree was identified within each of 5 compass subdirections (N, NE, E, SE, S, SW, W, and NW) that were randomly selected using an orientation generator; this design resulted in 15 total tree height measurements per site cluster (5 trees at each of the 3 plot locations). We used a TruPulse laser hypsometer to measure height, selecting 3 points on the trunk of each tree (horizontal angle from measurer, top, bottom) using a filter set for FARTHEST to prevent tree limbs from interfering with the measurement. To estimate horizontal percentage cover of ERC at each site, we defined 10 m-radius circular plots centered on the same 3 trees used as plot centers for tree height measurement, and we walked through the entirety of the circular plot to visually estimate the percentage of the plot horizontally covered by living ERC. The same observer performed all horizontal cover estimates to prevent sampling bias. Based on these measurements, we generated estimates of average tree height and horizontal cover across all sites in each ERC encroachment stage. We found that our sites still captured 3 distinct stages of ERC encroachment in early 2024 with regard to the average height of trees and the average percent horizontal coverage of trees (Stage 2 height: mean = 3.6 m, median = 3.5 m, range = 2.5 to 4.0 m, and cover: mean = 30.2%, median = 33.8%, range = 16.3% to 36.3%; Stage 3 height: mean = 5.3 m, median = 4.9 m, range = 4.4 to 6.3 m, and cover: mean = 55.2%, median = 55.0%, range = 40.0% to 58.8%; Stage 4 height: mean = 7.2 m, median = 6.6 m, range = 5.6 to 9.4 m; and cover: mean = 89.3%, median = 87.5%, range = 82.5% to 97.5%), and also that these stages were substantially different from open-grassland control sites used in the companion study (Fig. 3).

### Capturing Birds and Sampling Ticks

From mid-May to October of 2023, mist-netting was performed to collect ticks off live birds; sampling stopped in October due to constraints related to funding, staffing, and the academic calendar. We originally attempted to mist net in Stage 1 sites (open grasslands), but initial efforts were unsuccessful in capturing birds representative of grasslands due to the open vegetation structure and difficulty of setting up nets without causing birds to leave the area. We therefore focused only on the remaining 3 stages of encroachment, sampling at 3 sites in each of stages 2, 3, and 4 (ie 9 total sites sampled), where the presence of corridors between vegetation enabled successful bird captures. The 9 sites we used were: Stage 2—Lake Carl Blackwell, Fort Cobb State Park West, and American Horse Lake, Stage 3—Fort Cobb State Park East, OSU Research Range, and Boiling Springs State Park, Stage 4—Boiling Springs State Park, Fort Cobb State Park West and Canton WMA. Larval and nymphal tick instars are most common in late spring and mid-summer and are frequently found on birds during this period when most birds are breeding (Bouzek et al. 2013, Roselli et al. 2022). As *A. americanum* is the dominant tick species in our study area (Noden et al. 2022), we attempted to sample at each of the 9 sites once in late-May/early June corresponding to this species’ peak nymphal season (McClung et al. 2023); once in late July corresponding to its peak larval season; and once in late-August/early September, a period when this species is still highly active and abundant. This study design would have resulted in a total of 27 total collection days; however, due to weather constraints, we could only sample 2 times at 4 of the sites (ie 23 total collection days, with 7, 8, and 8, in Stage 2, 3, and 4 sites, respectively). The order of sites sampled was randomly generated before each sampling season, although minor alterations to the sampling schedule occurred in some cases due to weather.

During each sampling day, we captured birds using 6 to 8 mist nets (2.6 m in height, 12 m in length, 38 mm mesh, Avinet Inc., Dryden, NY) that were open between roughly sunrise and 10:30 AM, except when extreme heat (>32 °C) or precipitation caused us to close nets early. The net mesh size was selected to capture a wide range of passerine and near-passerine (eg woodpeckers and cuckoos) bird species, which make up the majority of birds that occurred in our study sites. All bird handling was permitted under a US Geological Survey federal bird banding permit (#23929), an Oklahoma Department of Wildlife Conservation permit (#W2304), and approved by an Institutional Animal Care and Use Committee at Oklahoma State University permit (#23-09-STW). Since little is known about which bird species carry ticks in ERC-encroached areas, any healthy and alert passerine or near-passerine caught in the net was examined for ticks. For all captured birds, we used a protocol previously developed in the same region (Roselli et al. 2020) to thoroughly check all body parts (head, neck, underwing, flanks, legs) for the presence of ticks by blowing or pushing feathers aside to check the skin of the bird. To prevent undue stress or injury, we released one bird without performing tick checks, and we also incidentally captured 3 birds that we did not have permits to handle (2 hummingbirds and 1 owl) and thus had to release immediately. Ticks were carefully removed from birds in the hand using needle-tipped forceps and placed immediately after collection in a 1.5 ml tube containing 70% ethanol. Ticks were associated with each bird and date of sampling on a data sheet, and we tallied total numbers of each tick species removed, as well as age of each. The body part from which we sampled each tick was also recorded in the field. All ticks from each bird were initially placed in a single tube in the field, but individual ticks were placed in separate tubes in the lab. Ticks were identified to species within 2 weeks of sampling using pictorial keys (Keirans and Litwak 1989, Keirans and Durden 1998, Coley 2015, Dubie et al. 2017). After identification, individual ticks were screened using previously published PCR protocols targeting 16S rRNA gene (TQ16S + 1F/TQ16S-2R) to confirm larval and nymphal tick identification (Halos et al. 2004, Crowder et al. 2010).

### Statistical Analysis

To address objective 1, we generated tabular summaries of the number of ticks found on each bird species, then calculated prevalence of infestation (total number of birds with at least one tick divided by total number of birds searched for ticks) and intensity of infestation (total number of ticks across all individuals divided by total number of infested individuals), with both metrics calculated across all birds combined as well as for individual bird species (Kahl et al. 2002). For objective 2 (formal analysis of the effect of ERC stage on tick infestation of birds), we first calculated site-level prevalences of tick infestation across all bird species (ie total number of birds of any species with at least one tick divided by total number of birds searched for ticks at the site) and intensities of tick infestation across all bird species (ie total number of ticks sampled from all birds divided by total number of birds with greater than one tick at the site). Because it is not possible to divide by zero, we excluded sites from the intensity of infestation calculation if no birds captured from the site were found to be infested with ticks. For the prevalence of infestation analysis, we used a generalized linear model which treated sites as replicates (*n* = 9) and ERC encroachment stage as a fixed effect, and that included a binomial distribution that treated the number of birds with greater than one tick as the number of “positive” outcomes out of a sample of binomial trials represented by the number of birds sampled. For the intensity of infestation analysis, we used a linear model (after determining that residuals of the response variable were distributed normally) that also treated sites as replicates and ERC stage as a fixed effect. For both analyses, significance of the effect of ERC stage was determined using a Likelihood ratio test that compared a model with the stage fixed effect to an intercept-only model (significance inferred at an alpha level of ≤0.05).

## Results

### Descriptive Summary of Prevalence and Intensity of Tick Infestation of Birds

We captured 144 birds and sampled 140 for ticks (excluding 4 birds that we released to avoid undue stress and/or for which we did not have permits to handle) over the course of the field season which represented 618 net hours of sampling across all sites. Only one individual bird was captured more than once, and it did not have ticks present on either of its two sampling occurrences. The most frequently sampled bird species was Northern Cardinal (*Cardinalis cardinalis*) (53 individuals) followed by Carolina Chickadee (*Poecile carolinensis*) (28) and Painted Bunting (*Passerina ciris*) (11). Regarding sex of the birds captured, 37.1% (52/140) were male 25.0% (35/140) were female, and 37.9% could not be sexed (35/140) (individuals in age-groups and/or of species that are sexually monomorphic).

We collected 90 total ticks from 8 different bird species (Table 2). Overall, 36 out of 140 sampled birds (25.7%) had at least one tick, and the average intensity of infestation among these birds was 2.5 ticks. Among bird species checked at least 5 times, Carolina Wren (*Thryothorus ludovicianus*) had the highest infestation prevalence (60%; 3/5), followed by Northern Cardinal (49.1%; 26/53). Under the same criteria, both species also had the highest intensities of tick infestation, with 2.67 (8/3) and 2.62 (68/26), respectively. These 2 species represented 21.4% (6/28) of all captures at Stage 2 sites, 40.0% (17/43) at Stage 3 sites, and 51.0% (35/69) at Stage 4 sites. Some bird species with high prevalence or intensity of infestation were only sampled 1 or 2 times (eg Brown Thrasher [*Toxostoma rufum*], Summer Tanager [*Piranga rubra*], and Swainson’s Thrush [*Catharus ustulatus*]); therefore, their infestation estimates must be interpreted with caution and may solely be an artifact of low sample sizes. The most common species of tick found on birds was *A. americanum* (52 individuals; 57.8% of all ticks), followed by *Haemaphysalis leporispalustris* (32 individuals; 35.5%), *Ixodes scapularis* (5 individuals; 5.5%), and *Amblyomma maculatum* (1 individual; 1.1%). All ticks sampled were either nymphs (*n* = 54; 60.0%) or larvae (*n* = 36; 40.0%). *A. americanum* and *I. scapularis* were sampled from multiple birds across multiple sites and days, with *I. scapularis* collected exclusively from Carolina Wren and Painted Bunting. All *H. leporispalustris* were sampled from one Stage 4 site on a single day in early October (only collected from Northern Cardinal), and the one *A. maculatum* was sampled from a Stage 4 site on a single day in mid-July (collected from a Painted Bunting).

**Table 2.** Tabulated summaries of ticks on each species of bird sampled. Includes: number of birds checked for ticks, number of birds with at least one tick, prevalence of infestation (the number of birds carrying at least one tick divided by total birds sampled), intensity of infestation (the average number of ticks on birds with at least one tick), and numbers of each tick species separated by species and life stage, for each bird species, for a study of tick infestation of birds in relation eastern redcedar encroachment in Oklahoma, USA in May to October 2023

					*Amblyomma americanum*		*Amblyomma maculatum*	*Ixodes* *scapularis*		*Haemaphysalis leporispalustris*	
Bird species	Birds checked	Birds with ticks	Prevalence of infestation	Intensity of infestation	Nymph	Larvae	Larvae	Nymph	Larvae	Nymph	Larvae
**Bewick’s Wren** (*Thryomanes bewickii*)	1	0	0								
**Black-and-white Warbler** (*Mniotilta varia*)	1	0	0								
**Blue-gray Gnatcatcher** (*Polioptila caerulea*)	1	0	0								
**Brown Thrasher** (*Toxostoma rufum*)	1	1	100 (1/1)	5 (5/1)	5						
**Brown-headed Cowbird** (*Molothrus ater*)	4	0	0								
**Carolina Chickadee** (*Poecile carolinensis*)	28	0	0								
**Carolina Wren** (*Thryothorus ludovicianus*)	5	3	60 (3/5)	2.67 (8/3)	4			1	3		
**Common Grackle** (*Quiscalus quiscula*)	6	1	16.6 (1/6)	1 (1/1)		1					
**Downy Woodpecker** (*Picoides pubescens*)	1	0	0								
**Field Sparrow** (*Spizella pusilla*)	1	0	0								
**Indigo Bunting** (*Passerina cyanea*)	4	1	25 (1/4)	2 (2/1)	2						
**Northern Cardinal** (*Cardinalis cardinalis*)	53	26	49.1 (26/53)	2.62 (68/26)	35	1				2	30
**Ovenbird** (*Seiurus aurocapilla*)	1	0	0								
**Painted Bunting** (*Passerina ciris*)	11	2	18.2 (2/11)	2 (4/2)	2		1	1			
**Red-eyed Vireo** (*Vireo olivaceus*)	3	0	0								
**Summer Tanager** (*Piranga rubra*)	2	1	50 (1/2)	1 (1/1)	1						
**Swainson’s Thrush** (*Catharus ustulatus*)	1	1	100 (1/1)	1 (1/1)	1						
**Tufted Titmouse** (*Baeolophus bicolor*)	7	0	0								
**White-eyed Vireo** (*Vireo griseus*)	5	0	0								
**Yellow-billed Cuckoo** (*Coccyzus americanus*)	2	0	0								
**Yellow-breasted Chat** (*Icteria virens*)	1	0	0								
**Yellow-rumped Warbler** (*Setophaga coronata*)	1	0	0								
**Totals**	140	36	25.7	2.5	50	2	1	2	3	2	30

### Comparison of Tick Infestation of Birds among Eastern Redcedar Encroachment Stages

**Fig. 4.**
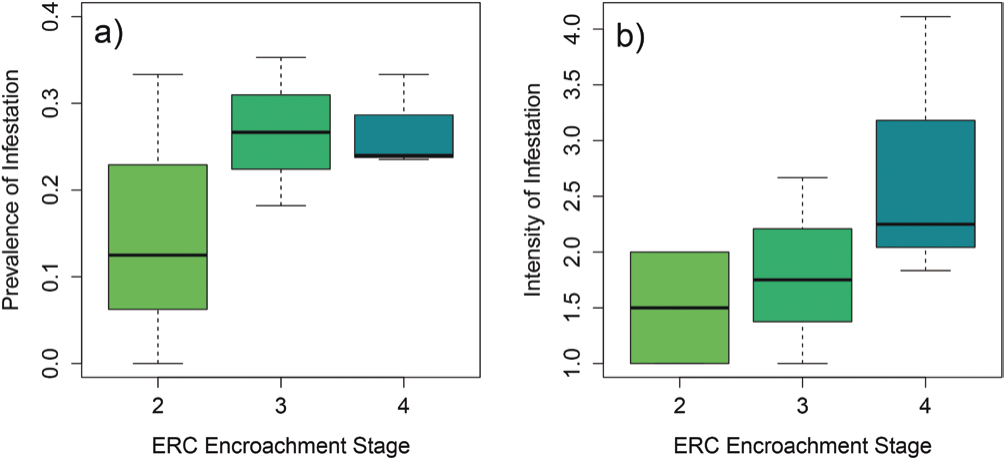
Box plots of: A) prevalence of tick infestation (# of birds sampled with at least one tick/total number of birds sampled) and B) intensity of tick infestation (average number of ticks on infested birds) collected from birds in 3 ERC stages across 9 sampled sites in Oklahoma, USA in May to June 2024. Each stage had 3 sampling replicates, except Stage 2 for intensity of infestation, since one site had zero birds collected with ticks. Overall group *P* values were calculated using a Walz *χ*^2^ Anova analysis between all stages; there was no significant effect of ERC stage on tick infection prevalence (*P* = 0.69) or intensity (*P* = 0.40).

Regarding differences in prevalence of infestation among stages of ERC encroachment, the average prevalence of birds with at least one tick was 0.18 for Stage 2 (range = 0, 0.33), 0.28 for Stage 3 (range = 0.18, 0.35), and 0.28 for Stage 4 (range = 0.24, 0.33). Regarding differences in intensity of infestation, the average number of ticks on infested birds was 1.5 for Stage 2 (range = 1, 2), 1.81 for Stage 3 (range = 1, 2.67), and 2.73 for Stage 4 (range = 1.83, 4.11). We found no statistically significant effect of ERC encroachment stage for prevalence of infestation (Likelihood-Ratio *χ*^2^ test = 0.74, Degrees of Freedom = 2, *P* = 0.69) and intensity of infestation (Likelihood-Ratio *χ*^2^ test = 2.17, Degrees of Freedom = 2, *F*-value = 1.12, *P* = 0.40) (Fig. 4).

## Discussion

We found that roughly one-quarter of all birds captured within ERC-encroached grasslands were infested with ticks, a higher prevalence of infestation than in most previous studies of birds in the United States (Loss et al. 2016). We also found that Northern Cardinal and Carolina Wren, both nonmigratory resident species, appear to be the greatest avian contributors to maintaining abundance of ticks in areas experiencing ERC encroachment. Despite this high tick infestation prevalence, there were no significant differences in prevalence and intensity of tick infestation of birds across the 3 stages of ERC encroachment we studied, which indicates that many birds carry ticks even in the earliest stages of encroachment. Overall, these results indicate that birds are likely contributing to the maintenance of tick populations, and therefore, to the transmission of tick-borne pathogens in grasslands of the Great Plains regions that are experiencing ERC encroachment.

### Prevalence and Intensity of Tick Infestation in Association with Eastern Redcedar Encroachment

Across all sites, 25.7% of birds were infested with at least one tick. This prevalence of infestation is 5 times higher than the overall prevalence of infestation (5.1%) calculated based on 11 different studies in a review of tick infestation of birds in North America (Loss et al. 2016), and similar to the 24.2% from an urban focused study in this region (Roselli et al. 2022). Two species (Carolina Wren and Northern Cardinal) known to occur commonly in ERC-encroached areas (Holthuijzen and Sharik 1985, Horncastle et al. 2004, Heinen and O’Connell 2009) had the highest prevalence of tick infestation (60% and 49%, respectively) among species we sampled. The ground-foraging behavior of these species (De Graaf et al. 1985), which has been shown to be associated with higher tick infestation levels—likely due to a greater probability of interacting with and acquiring ticks (Mitra et al. 2014, Loss et al. 2016, Roselli et al. 2022)—may account for the high tick loads of these species. Further, the review of North American studies that sampled ticks from birds ranked Northern Cardinal first and Carolina Wren fourth among all bird species in in terms of prevalence of infestation (Loss et al. 2016). Thus, these and perhaps other bird species that occur in ERC and behave in ways that increase their chances of obtaining ticks, may contribute more to the maintenance of tick populations in ERC-encroached areas than species with different life histories, such as those that forage in tree canopies. Further, the abiotic conditions altered by ERC encroachment (eg shade and higher humidity than grasslands) (Caterina et al. 2014, Zou et al. 2015, 2018, Acharya et al. 2017) may provide an ideal habitat for the “nidus of infection” where birds, ticks, and pathogens interact (Noden et al. 2021).

Discovering a high prevalence of tick infestation of birds suggests birds are moving ticks into, out of, and among areas experiencing ERC encroachment. Range expansion of *A. americanum* and other ticks across Oklahoma (Barrett et al. 2015, Mitcham et al. 2017), and in other states in the Great Plains experiencing ERC encroachment (Cortinas and Spomer 2013, Maegli et al. 2016, Luedtke et al. 2020), may occur partially as a result of birds carrying ticks in association with ERC. The presence of a Central American tick, *Amblyomma longirostre*, in southeast Oklahoma can only be explained by a tick dropping off a migratory bird during or shortly after completing its northbound spring migration (Noden et al. 2015), and several instances of migratory birds bringing Neotropical ticks into temperate regions of North America have been documented (Hamer et al. 2012, Cohen et al. 2015, Karim et al. 2024). If ticks transported by birds can establish populations (Hasle 2013, Buczek et al. 2020), then birds may supplement populations of these ticks and, therefore, the presence of tick-borne pathogens in states experiencing high levels of ERC encroachment. Although we confirmed that a high proportion of birds carry ticks in ERC-encroached areas, it remains unknown what the relative contribution of birds is to introducing and maintaining tick populations compared to other tick bloodmeal hosts that use ERC, such as mice (Horncastle et al. 2005) and deer (Barrett et al. 2015, Tsao et al. 2021). Further research that samples ticks from a wide diversity of wildlife species, including mammals, birds, and reptiles, would increase clarity about the roles of these wildlife groups in the ecology of ticks and tick-borne diseases.

Some unique observations were made regarding the species and life stages of ticks found on birds in this study. Perhaps most notably, 3 Carolina Wrens and one Painted Bunting were infested with *I. scapularis* nymphs and larvae. Birds commonly carry this tick species in the northern and eastern parts of North America (Ogden et al. 2008, Brinkerhoff et al. 2011, Hamer et al. 2012, Cumbie et al. 2021), but this is the first recovery of *I. scapularis* on a bird in Oklahoma. In parts of the United States where the northern clade of *I. scapularis* is present, larval and nymphal ticks feed on *Borrelia burgdorferi*-infected rodents (Devevey and Brisson 2012) but are also commonly found on birds (Anderson et al. 1987, Stafford et al. 1995, Loss et al. 2016). The larvae and nymphs of the southern clade of *I. scapularis* are thought to primarily feed on reptiles (Garvin et al. 2015), thereby eliminating the risk of *B. burgdorferi* transmission (Kollars et al. 1999, Garvin et al. 2015, Ginsberg et al. 2021). Studies exploring this phenomenon in the Southern US did not find larval or nymphal *I. scapularis* ticks in the leaf litter (Dubie et al. 2018, Tietjen et al. 2019), and another study in the same region that included collection of ticks from birds did not find *I. scapularis* (Roselli et al. 2022). Our observation is important because it supports that the southern clade of *I. scapularis* can be transported by birds, just like their northern counterpart, and is not limited to feeding on reptiles, which suggests a novel aspect to the ecology of this tick that may be facilitated by ERC encroachment and have implications for the disease ecology of Lyme disease, Anaplasmosis, and Powassan virus in the region.

### Tick Infestation Prevalence and Intensity in Relation to ERC Encroachment Stages

Our analyses of the effect of ERC encroachment stage on prevalence and intensity of infestation indicate that tick infestation of birds is comparably high across all 3 encroachment stages we evaluated, a result that contradicts our prediction that infestation would increase with each subsequent encroachment stage. Combined with our overall high estimate of infestation prevalence, this result implies that a high proportion of birds are transporting ticks in association with ERC, regardless of the stage of encroachment. High infestation levels across all stages may be explained by larger-scale characteristics of landscapes and regions, for example, the large-scale amounts of different land cover types, which influence populations of ticks, hosts, and pathogens (Allan et al. 2003, Lambin et al. 2010, Diuk-Wasser et al. 2021), potentially resulting in relative homogeneity of tick populations across the landscape. Another possible explanation is provided by a companion study that sampled ticks from vegetation in the same study area (Propst 2025). That study found that, for most tick species and life stages, abundance was significantly greater in the earliest stage of encroachment with ERC trees present (Stage 2) compared to open grassland. Abundance differences among stages 2, 3, and 4, varied by tick species, life stage, and trapping method; however, for nymphs of *A. americanum* (the species most frequently found on birds in this study) that were collected by flagging (a trapping method that approximates abundance of host-seeking ticks), abundances were similarly high between stages 2 and 4. This pattern of a high abundance of host-seeking ticks across different encroachment stages could explain the lack of differences in tick infestation among stages in the current study. Additionally, although we only analyzed infestation of all birds by all tick species combined, there could be species-specific patterns of infestation—both for individual bird and tick species—in relation to the different stages of encroachment. Such species-specific patterns might occur as a result of interactions between the unique ecological traits of individual bird and tick species and the abiotic conditions, vegetation, and other factors that change as encroachment progresses. We were unable to evaluate this possibility due to the relatively small numbers of birds and ticks in our sample, but future research could evaluate such species-specific patterns.

### Limitations and Future Research Directions

We encountered limitations that should be considered when interpreting our results and that highlight promising directions for future research. One limitation was that we only collected birds over a single field season and at 9 study sites. This limited the number of birds and bird species that we were able to sample and collect ticks from, as well as the statistical power for our analyses of the effect of ERC encroachment stage. Additionally, our approach of using ground-based mist nets that only captured birds within 2.6 m of the ground—and of using a 38 mm net mesh size, which does not allow capture of bird species larger than doves and jays—prevented us from sampling ticks from all bird species that occurred in our sites. Further research targeting larger birds (eg Wild Turkeys [*Meleagris gallopavo*] and American Crows [*Corvus brachyrhynchos*]) and species that primarily occur higher in the tree canopy (eg vireos and tanagers) would yield valuable insight into prevalences and intensities of tick infestation for the entire bird community. Furthermore, although a multiseason study would generate a larger sample of birds and allow increased replication of each ERC encroachment stage, our single-year study nonetheless provided data to illustrate interesting patterns regarding the role of birds in carrying ticks (ie the high overall infestation prevalence that was consistent among encroachment stages). Another limitation is that, after initial unsuccessful attempts to capture birds in open grasslands, we were unable to evaluate tick infestation of birds in sites representing a grassland control, and therefore, could only make comparisons between areas experiencing different levels of ERC encroachment. Mist-netting is often ineffective in open grasslands since capture of birds usually requires placing nets in corridors between trees and shrubs. Future studies seeking to capture birds in grasslands in order to make comparisons between grasslands and areas experiencing WPE could utilize A-shaped nets (Martin 1969) and a team of observers to help funnel birds across the field and flush them into nets.

Lastly, the scale of our study sites was smaller than the scale of movements and home ranges of some of the bird species we sampled, and sites within clusters were likely more similar to each other than to sites in other clusters, leading to nonindependence and spatial autocorrelation concerns. We were prevented from taking a larger-scale approach due to the substantial challenge of receiving permission to access large areas of land that contain all ERC encroachment stages (97% of land in Oklahoma is privately owned [York and Jager 2021]), but this approach may be possible in regions with a larger proportion of public land. We also were unable to account for nonindependence using a random effect for site cluster due to the limited sample size of sites and associated model convergence issues. Further research that captures and replicates the various stages of WPE at broader, landscape-level scales, could address this limitation and increase understanding of how the role of birds in carrying ticks changes in different landscapes experiencing different stages of encroachment.

## Conclusions

Eastern redcedar encroachment is greatly impacting grasslands of the US Great Plains, including through its adverse effects on livestock production, wildfire risk, vegetation and wildlife diversity, and water availability (Barger et al. 2011, Starks and Moriasi 2017, Zou et al. 2018). Along with research showing that ERC is associated with increased abundance of ticks (Noden et al. 2021, Propst 2025), our study, which illustrates that a high proportion of birds carry ticks in ERC-encroached areas, further highlights that this encroaching tree may be increasing risk of tick-borne disease such as Spotted Fever Rickettsiosis and Ehrlichiosis (Biggs et al. 2016). Our results are associational, and we cannot confirm whether birds introduce ticks into ERC-encroached areas or if ticks are already present and birds are only picking them up in ERC. Nevertheless, this study provides compelling evidence that, at a minimum, birds are likely moving ticks from areas of existing ERC encroachment into other encroached areas and adjacent un-encroached habitats. Further, because we found that a high proportion of birds carry ticks even in the earliest stage of ERC encroachment, mirroring results of a companion study that collected ticks from vegetation, we provide additional evidence that the most effective way to limit expansion of tick populations is to prevent ERC encroachment before it starts or in early stages using approaches like prescribed fire and mechanical removal (Twidwell et al. 2021). This and additional research evaluating how the global phenomenon of WPE affects the ecology of ticks and tick-borne diseases, including the role of wildlife in carrying ticks, will inform land management and public health efforts seeking to reduce disease risk.
